# Impact of a training program on hospital pharmacists' patient-centered communication attitudes and behaviors

**DOI:** 10.1016/j.rcsop.2023.100325

**Published:** 2023-08-26

**Authors:** Yew Keong Ng, Noraida Mohamed Shah, Timothy F. Chen, Navin Kumar Loganadan, Shue Hong Kong, Yi Yun Cheng, Siti Shahida Md Sharifudin, Wei Wen Chong

**Affiliations:** a Centre of Quality Management of Medicines, Faculty of Pharmacy, Universiti Kebangsaan Malaysia, Jalan Raja Muda Abdul Aziz, 50300 Kuala Lumpur, Malaysia; b School of Pharmacy, Faculty of Medicine and Health, The University of Sydney, NSW 2006, Australia; c Department of Pharmacy, Hospital Putrajaya, Ministry of Health, Pusat Pentadbiran Kerajaan Persekutuan Presint 7, 62250 Putrajaya, Malaysia; d Department of Pharmacy, Universiti Kebangsaan Malaysia Medical Centre, Jalan Yaacob Latif, Bandar Tun Razak, 56000 Kuala Lumpur, Malaysia; e Department of Pharmacy, Hospital Ampang, Ministry of Health, Jalan Mewah Utara, Taman Pandan Mewah, 68000 Ampang Jaya, Selangor, Malaysia; f Department of Pharmacy, Hospital Kuala Lumpur, Ministry of Health, Jalan Pahang 50586, Kuala Lumpur, Malaysia

**Keywords:** Patient-centered care, Communication skills training, Motivational interviewing, Four Habits Model, Concordance

## Abstract

**Background:**

Effective communication that integrates the value of patient-centered care is important in healthcare encounters. Communication skills training (CST) has been indicated as effective in improving patient-centered communication behaviors. However, there is a paucity of studies on the impact of CST among Malaysian hospital pharmacists.

**Objective:**

This study aimed to evaluate the effects of a patient-centered CST program on patient-centered communication scores, communication self-efficacy, and attitudes toward concordance among pharmacists in public hospitals.

**Methods:**

A communication skills training (CST) program was conducted among hospital pharmacists. This training intervention was developed based on patient-centered communication frameworks and techniques, namely the Four Habits Model and motivational interviewing. A pre-test/post-test quasi-experimental design was implemented for the evaluation. Pharmacists underwent pre-test/post-test audiotaped simulated consultations and completed questionnaires, including the Revised United States–Leeds Attitudes Toward Concordance scale (RUS-LATCon) and Communication Self-Efficacy scale. The Four Habits Coding Scheme (FHCS) was used to evaluate patient-centered communication scores from the audiotapes, and the Wilcoxon signed-rank test was used to analyze for differences in the pre- and post-intervention scores.

**Results:**

A total of 38 pharmacists from four tertiary hospitals participated in this study and completed the pre-test. However, due to the impact of COVID-19, only 23 pharmacists completed the post-test data collection. Improvements were noted in the FHCS scores post-training, including items related to exploring patients' concerns, acceptability, and barriers to treatment. Based on the questionnaire, there was an improvement in recognizing patients' needs and potential medication uncertainty and an increase in the overall communication self-efficacy scores after the training.

**Conclusions:**

CST may help improve the adoption of patient-centered communication in pharmacists' consultations with patients.

## 1 Introduction

Effective communication is crucial in healthcare to ensure accurate information exchange between patients and healthcare providers, including pharmacists.^1^^,^^2^ The World Health Organization has emphasized the important role of pharmacists as communicators in facilitating rapport and information exchange between physicians and patients.^3^ Furthermore, effective communication is integral to pharmacists' medication management responsibilities, which include patient counseling and education. It significantly contributes to patients' knowledge and beliefs about medications, leading to improved treatment satisfaction and enhanced medication adherence.^4^ In addition, effective communication can lead to better patient health literacy and increased self-efficacy in disease management.^5^, ^6^, ^7^

Beyond its role in information exchange, effective communication serves as an important enabler of patient-centered care (PCC).^8^ Patient-centered communication involves understanding the patient's subjective experience and unique psychosocial context, being responsive to their concerns and needs, and actively involving them in healthcare discussions.^9^^,^^10^ This is in contrast to healthcare provider-centered communication, where providers' technical skills and knowledge predominate, reflected through behaviors such as direct and closed questioning of the patient and giving instructions.^11^ Prioritizing patient-centered communication ensures a more collaborative and empathetic approach to healthcare that can lead to better patient outcomes.^12^

Communication skills training (CST) has been proposed as an effective approach for improving patient-centered communication skills among healthcare providers, including pharmacists.^11^^,^^13^ Numerous studies have reported positive outcomes following CST, such as increased self-efficacy in communication, which correlates with a more effective application of patient-centered communication techniques during consultations.^14^, ^15^, ^16^, ^17^ Studies have also found more frequent use of empathetic responses and socioemotional aspects in pharmacists' counseling post-CST, underscoring the potential of CST to improve pharmacist-patient interactions.^18^^,^^19^

Incorporating communication frameworks into CST training can provide structure and aid practitioners in applying patient-centered communication during patient encounters. Various frameworks have been proposed, including the Four Habits Model (FHM), Calgary-Cambridge model, AIDET (Acknowledge-Introduce-Duration-Explanation-Thank you), 5A (ask, advise, agree, assist, arrange), and SBAR (Situation-Background-Assessment-Recommendation).^20^, ^21^, ^22^, ^23^, ^24^ Among them, the FHM has demonstrated better compatibility with pharmacists' encounters.^25^, ^26^, ^27^ The Four Habits Model (FHM) was developed for training in a continuing education context and consists of four domains, called habits, which are structured but interrelated in nature.^20^ Studies utilizing the FHM in healthcare provider CST have demonstrated increased patient-centered communication scores after training.^26^^,^^28^ Additionally, this model has been validated through video recordings of actual encounters and adapted for pharmacy consultations.^25^^,^^26^^,^^29^ Furthermore, the FHM organizes patient-centered communication skills in a more efficient and logical structure, making it easier to recall and for pharmacists to self-practice in busy settings.^26^

Another widely used patient-centered communication technique, namely motivational interviewing (MI), has also been frequently used in communication training. This technique is particularly useful for exploring and addressing patients' ambivalence toward behavioral changes.^30^ Using MI communication techniques, healthcare providers, including pharmacists, can effectively plan and direct patients' motivation for change, develop rapport with them, and formulate plans to achieve behavioral changes.^31^^,^^32^ MI training studies among providers have demonstrated increased healthcare satisfaction among patients and sustained behavioral changes, including improved medication adherence and lifestyle changes (e.g., dietary restrictions).^13^^,^^33^^,^^34^ Moreover, MI has demonstrated suitability for implementation by various healthcare providers, including pharmacists, making it a valuable strategy for enhancing medication adherence and clinical outcomes for patients with chronic diseases.^35^

In Malaysia, medication therapy adherence clinic (MTAC) services are provided by hospital pharmacists to monitor patients' medication adherence, provide counseling, and reinforce lifestyle modifications to manage their chronic diseases.^36^^,^^37^ It also serves as a platform for pharmacists to understand patients' concerns and motivations for continuing their drug therapy,^36^ highlighting the need for patient-centered communication to improve patients' medication-taking behavior. However, few studies have evaluated MTAC pharmacists' communication behaviors and the impact of CST on patient-centered communication. Existing communication-related studies were mainly conducted among Malaysian pharmacy students, which have shown improvements in students' communication skills after CST.^38^^,^^39^ This represents an important gap in research that highlights the need for a CST specifically tailored to practicing pharmacists. Such training would equip them with the necessary communication skills to provide effective patient-centered care in disease management.

Thus, this study aimed to evaluate the effects of a patient-centered CST program on patient-centered communication scores, self-reported communication self-efficacy, and attitudes toward concordance among MTAC pharmacists in public hospitals.

## 2 Methods

### 2.1 Study design and setting

This study employed a quasi-experimental design involving a single-group pre- and post-test intervention among pharmacists at one teaching hospital and three tertiary hospitals in Malaysia. The adoption of this study design was based on its suitability as an initial guide for future CST research. Additionally, the quasi-experimental design was deemed appropriate upon consideration of viability and practicality issues.

**Fig. 1:**
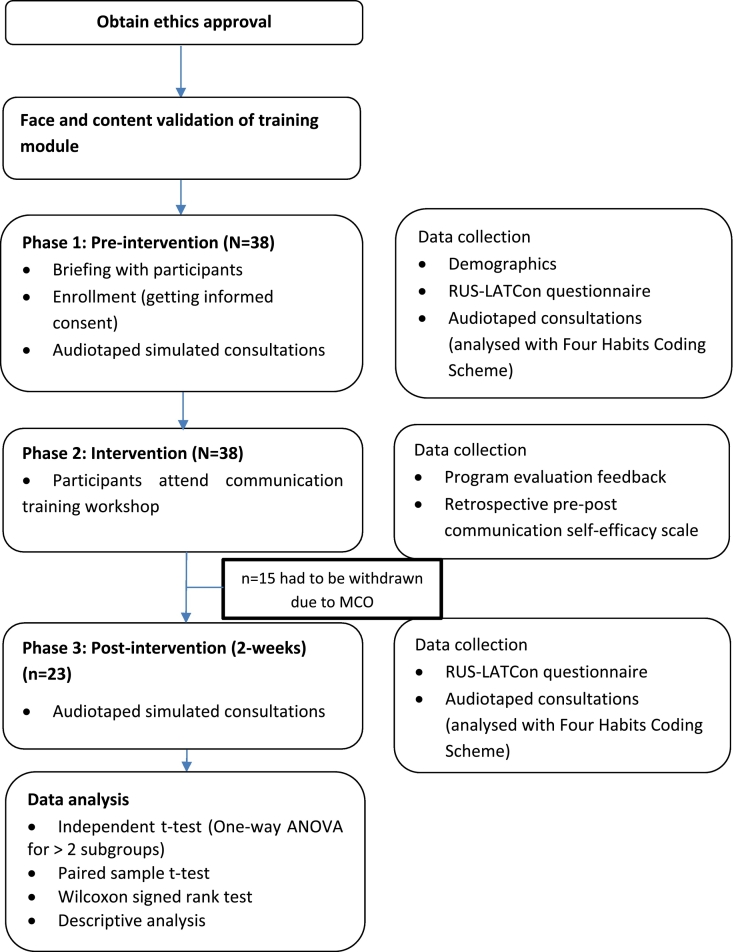
Flowchart of study. ANOVA: Analysis of Variance. MCO: Movement Control Order (implemented due to COVID-19 pandemic). RUS-LATCon: Revised United States Leeds Attitudes toward Concordance scale.

The study was divided into three phases (Fig. 1). The first phase involved recruitment and pre-intervention data collection that consisted of a pre-intervention questionnaire and simulated consultation. The second phase was the intervention phase, in which participants underwent a one-day communication training workshop. The third phase was the post-intervention phase, consisting of a second round of simulated consultation and administration of a post-intervention questionnaire.

### 2.2 Participant recruitment

Eligible participants were recruited with the help of the site co-investigators (NKL, SHK, YYC, SSS) from the four locations. The site co-investigators facilitated the recruitment of eligible participants through e-mail and face-to-face recruitment. A briefing session by YKN was also arranged to facilitate the recruitment. During the briefing, an overview of the study was presented, and participants had the opportunity to ask questions for clarification. A recruitment poster, agenda, and participant information sheets were distributed among prospective participants throughout the recruitment process. Pharmacists were eligible to participate if they were at that time involved in providing MTAC services to patients receiving warfarin and/or patients with type 2 diabetes. These specific MTAC services were selected because a higher number of pharmacists were involved at the site in providing these services, and both required long-term management of medication adherence. Pharmacists were excluded if they were unable to participate for various reasons (e.g., on leave or applying for transfer).

Using G*Power (v3.1.9.4) calculator by Faul et al. (2007),^40^ 15 hospital pharmacists were deemed sufficient for this study based on the following information: (a) difference between two dependent means (matched pair), (b) large effect size dz. = 0.8, (c) alpha = 0.05, (d) power = 0.80, and (e) repeated measures on two timeframes. With this information, a total of 30 hospital pharmacists currently providing MTAC services were targeted to be included in this study after accounting for possible dropouts, no-shows, and scheduling issues.

### 2.3 Development of training module and program

A training program was planned and developed, consisting of a series of seven consecutive steps adapted from Brown et al. (2010): (1) conducting a literature search, (2) holding meetings to reach a consensus, (3) developing a framework, (4) producing training materials, (5) creating scenarios, (6) making revisions and adaptations, and (7) conducting assessments.^41^ First, a literature search on CST was conducted to identify effective training elements and theoretical communication frameworks used. Second, meetings were held within the research team to reach a consensus on the suitable communication frameworks to be used. Subsequently, after comparing each model, the FHM framework and MI approach were deemed suitable and were adapted for this study. The FHM was selected because of its emphasis on PCC aspects and previous findings supported its application in the pharmacist-patient encounters.^25^^,^^26^ The MI technique was selected based on its relevance in the MTAC pharmacy setting, which focused on improving and motivating patient adherence to medications.

The fourth step included the development of a training module, whereby it was developed based on the core aspects of the FHM and MI. As part of the fifth step, SP scenarios were created for training assessment purposes. As part of the sixth step, all adapted materials and modules were discussed and reviewed by the academic research team before the training to ensure that they were valid and appropriate. The developed training module was then sent to practitioners who were also the site investigators in this study. Interviews were then conducted with the practitioners to evaluate the face and content validity of the module. The last step involved conducting the training program to test the developed module. The impact of the training program was evaluated through pre- and post-intervention data collection, which involved audiotaped simulated consultations and questionnaires. Further details regarding these methods are provided below.

The training program was an 8-h workshop that included sessions by four invited speakers who shared their experiences and expertise. The speakers comprised academicians, MTAC pharmacists, and a dietician, all with extensive knowledge in patient-centered communication training. The training consisted of four sessions that combined didactic lectures with interactive group discussions based on case scenarios, as described in Table 1.

**Table 1:** Overview of training sessions.

Session 1: Patient-centered care (PCC) in pharmacy
This session consisted of a didactic lecture that aimed to introduce the general concepts of PCC and its application in providing medication therapy adherence clinic (MTAC) services to participants. Based on the integrative PCC model by Scholl et al. (2014),^8^ participants were taught how patient-centeredness may be integrated into patient–pharmacist communication. Participants were also taught how these approaches may empower and allow patients to be actively involved in their treatment-related decision-making.
Session 2: Motivational interviewing (MI) in medication-taking
This session introduced the concept of MI and its application during communication with patients. MI is an evidence-based approach through a collaborative, patient-centered, directed form of conversation to explore patients' reasons for changes in their healthcare-related behaviors.^31^ Participants were also taught about MI techniques (e.g., OARS) and underwent practice scenario exercises related to diabetes and warfarin management. The practice case scenarios consisted of patients' non-adherence to insulin or warfarin, and the participants were divided into groups to discuss patient-centered communication strategies based on MI to address these issues.
Session 3: Empowering patients with type 2 diabetes to attain glycemic targets: the role of diabetes MTAC pharmacists
This session introduced the role of pharmacists in empowering patients with diabetes to achieve their glycemic targets. Participants were presented with real-life practice examples and strategies to overcome non-adherence issues common among patients with diabetes. It was emphasized that each patient manages their disease differently and that providing a tailored approach is important for attaining patients' therapeutic goals.
Session 4: Patient-centered communication and the FHM
The Four Habits Model (FHM) was introduced to the participants in this session. In addition, findings of previous studies related to the important aspects and factors influencing PCC in pharmacy were shared with the participants.^51^ This session also involved interactive case practice examples integrating the application of PCC, MI, and the FHM to reinforce participants' understanding and aid implementation in their practice.

### 2.4 Data collection

The data collection for the pre-intervention phase (Phase 1) involved one-to-one simulated MTAC consultations with simulated patients (SPs) and a questionnaire on participants' demographic details and an adapted Revised United States Leeds Attitudes Toward Concordance (RUS-LATCon) scale. Immediately after the training (Phase 2), the participants completed a post-workshop questionnaire consisting of program evaluation feedback and a retrospective pre–post communication self-efficacy scale. Approximately two weeks after the training (Phase 3), participants underwent another audiotaped face-to-face simulated consultation with SPs to evaluate the effectiveness of the training intervention. Additionally, a post-test questionnaire similar to the Phase 1 pre-intervention RUS-LATCon was completed.

#### 2.4.1 Simulated patient assessments and the Four Habit Coding Scheme (FHCS)

Simulated patient assessments were conducted to assess the effectiveness of the communication training intervention. The use of SPs has been recognized as a valid and reliable method for evaluating the effectiveness of communication interventions.^11^ Two scenarios were developed for diabetes and warfarin management, guided by best practice approaches for setting objectives and realism/fidelity, and reviewed by experienced practitioners and academics for validity.^42^^,^^43^ The objectives were set to evaluate participants' communication skills; thus, the case scenarios were developed for participants to address issues related to medication-taking behaviors during a typical MTAC counseling session. Participating pharmacists were expected to elicit patients' concerns about their medications and reasons for non-adherence issues, as well as provide appropriate counseling in a patient-centered manner. To enhance the scenarios, the expert panel suggested the addition of patient social history, patient knowledge level, and personal behaviors during the encounters, all of which were included. Before data collection, the SPs received training to ensure consistency in their performance and to identify any potential oversights or ambiguities in the scenarios.^42^

Appointment schedules for the simulated consultations were set by the co-investigators at each site for all study participants before and after the training. Each participant was allocated 10 min for the simulated consultations, and all interactions were audiotaped for analysis. To assess patient-centered communication performance, the Four Habits Coding Scheme (FHCS) was adapted and applied to the audiotaped consultations. The FHCS was originally developed and validated as a reliable tool for assessing providers' communication behaviors based on the Four Habits Model (FHM).^29^ Permission was sought from the original authors to use this scale for the purpose of data analysis. The coding scheme consisted of 23 items organized into the “Four Habits,” namely ‘invest in the beginning,’ ‘elicit the patient's perspectives,’ ‘demonstrate empathy,’ and ‘invest in the end.’ The items were scored on a 5-point scale from 1 (not very effective) to 5 (highly effective) with 3 as a mid-point score, and all scores were summed across all items.^29^ Two items (i.e., items 1A: show familiarity with patient and 3D: display effective nonverbal behavior) were not included, as the SP scenarios were set as the patients' first encounter with pharmacists (thus, pharmacists were not familiar with the patients) and nonverbal behavior could not be fully determined from the audio recordings alone. The FHCS has a reference codebook, which outlines the description of each item and how to score each item.^29^

The audio-taped consultations were coded by the first author (YKN), and a random sample of 10 audio-recordings (five from pre-test and post-test each) were selected and re-coded to assess intra-rater reliability. Overall, the intraclass coefficient was 0.75, and the interclass coefficient for each habit group ranged from 0.63 to 0.92, which were considered satisfactory.

#### 2.4.2 Revised United States Leeds Attitudes Toward Concordance (RUS-LATCon)

The RUS-LATCon was developed by Flagg (2010), which measures healthcare providers' attitudes toward concordance during their clinical encounters with patients.^44^ Concordance is a patient-centered approach defined as the agreement made after a negotiation between healthcare providers and patients, while considering the beliefs and wishes of patients on whether, when, and how treatment regimens are to be followed.^45^ This instrument was adapted in a previous study to suit the pharmacy context and used in this study to measure pharmacists' attitudes toward concordance before and after the training.^37^ This instrument consists of 14 questions measured on a 5-point Likert scale ranging from “strongly disagree” to “strongly agree”.

#### 2.4.3 Program evaluation feedback

The program evaluation feedback, administered to participants immediately after the training, measured their satisfaction using nine items on a scale of 1 (=very dissatisfied) to 5 (=very satisfied). The items included ratings for all sessions of the program, fulfillment of the program objectives, overall workshop rating, and program organization. The participants were also requested to provide a rating, from 1 (=not important at all) to 5 (=very important), in response to a retrospective pre–post question on how important they perceived CST to be before and after the training. Additionally, three open-ended questions gathered feedback on the most useful training component for practice, suggestions for future program improvement, and any additional comments.

#### 2.4.4 Retrospective pre–post communication self-efficacy scale

Immediately after the training, a retrospective pre–post communication self-efficacy scale was administered to participants. This scale consisted of 10 questions measuring participants' confidence in completing communication tasks, rated on a scale of 1 (=very uncertain) to 10 (=very certain). Self-efficacy is based on the theory by Bandura (1997) on one's confidence in performing a certain task: in this case, the communication task during the MTAC.^46^ The questions and format of the questionnaire were adapted from several self-efficacy studies that are relevant to the pharmacy context.^14^^,^^15^^,^^47^^,^^48^ A retrospective pre–post questionnaire format was implemented to reduce response shift bias. This bias is common in a traditional pre-test/post-test format whereby an underestimation of the effectiveness of the program may occur or participants may overestimate their confidence rating before attending the training, leading to the acquisition of inaccurate results.^49^^,^^50^

### 2.5 Data analysis

IBM Statistical Package for Social Sciences (SPSS®) v22 was used for the data analyses. Descriptive analyses were conducted to evaluate the demographic profiles and pre-test scores of the FHCS. Data are expressed as mean ± standard deviation (SD) unless stated otherwise. A value of *p* < 0.05 was considered statistically significant for all analyses. Written feedback from the participants in the post-workshop questionnaire was also described.

Data from the RUS-LATCon and communication self-efficacy fulfilled the normality assumption for parametric testing. As such, paired-sample *t*-tests were used to determine significant differences between the pre- and post-intervention scores of the RUS-LATCon and self-reported communication self-efficacy. Since the FHCS scores did not fulfill normality assumptions, nonparametric tests, such as the Wilcoxon signed-rank test, were used to analyze differences in the pre- and post-intervention FHCS scores.

### 2.6 Ethical considerations

The study was approved by the Research Ethics Committee of Universiti Kebangsaan Malaysia (Ref. No: PPI/111/8/JEP-2019-624) and Medical Research and Ethics Committee, Ministry of Health Malaysia (Ref No: NMRR-19-2522-50,414 (IIR)). All participants provided written informed consent.

## 3 Results

### 3.1 Participant demographics

A total of 38 pharmacists participated in both Phase 1, which involved collecting pre-intervention data, and Phase 2, which consisted of the training program (intervention phase). However, due to the impact of COVID-19 pandemic, only 23 pharmacists were able to complete the post-test data collection (Phase 3) (Fig. 1).

The participants' demographics are summarized in Table 2. The mean age of the participants was 31.76 (SD = 2.97) years; most of the participants were female (*n* = 33, 86.8%). In terms of ethnicity, there were the same number of Malay (*n* = 17, 44.7%) and Chinese (n = 17, 44.7%) participants, followed by Indians (*n* = 4, 10.5%). Only two of the pharmacists held a Master's degree qualification, while the others had a Bachelor's degree qualification. The number of participants recruited from the warfarin and diabetes mellitus MTAC groups was equal (*n* = 19, 50% each). Most of the participants had <5 years of experience practicing in MTACs (*n* = 21, 55.3%) and spent <5 h per week in MTACs (*n* = 32, 84.2%).

**Table 2:** Sociodemographic of pharmacists at baseline with FHCS scores (*N* = 38).

Descriptive characteristics	Frequency (N = 38)	Percentage, %	Overall FHCS baseline score, Mean (SD) (N = 38)	Parametric test p-value for baseline score between demographics
Gender				0.003^a^, ⁎
Male	5	13.2	3.48 (0.73)	
Female	33	86.8	2.54 (0.60)	
Age (years old)				
Mean (SD)	31.76 (2.97)			
Median (IQR)	32 (30–34)			
Ethnicity				0.90^b^
Malay	17	44.7	2.64 (0.64)	
Chinese	17	44.7	2.72 (0.74)	
Indian	4	10.5	2.56 (0.81)	
Highest academic qualification				0.08^a^
Bachelor's degree	36	94.7	2.62 (0.66)	
Master's degree	2	5.3	3.50 (0.84)	
Type of MTAC				0.58^a^
Warfarin MTAC	19	50	2.73 (0.51)	
Diabetes MTAC	19	50	2.60 (0.83)	
Years of experience as a practising pharmacist				0.97^a^
<5 years	21	55.3	2.66 (0.79)	
>5 years	17	44.7	2.67 (0.55)	
Hours spent in clinic consultation per week				0.77^a^
<5 h	32	84.2	2.68 (0.72)	
5–10 h	6	15.8	2.59 (0.50)	

a Independent *t*-test.

b One-way ANOVA.

⁎ *p* < 0.05.

As the baseline mean FHCS scores fulfilled the normality assumption, parametric tests (independent *t*-test and one-way analysis of variance) were used to determine significant differences across the demographics (Table 2). At baseline (*n* = 38), only gender revealed a significant difference, whereby men (mean (SD) = 3.48 (0.73)) scored higher than women (mean (SD) = 2.54 (0.60); *p* < 0.05).

### 3.2 Impact of training on patient-centered communication scores (pre-intervention versus post-intervention scores, *n* = 23)

Table 3 summarizes the FHCS scores. A good internal reliability (Cronbach's alpha) was obtained (pre-intervention α = 0.87, post-intervention α = 0.86). In general, the baseline scores for most items in the FHCS were less than the mid-score of 3. In Habit 1, only one item, “usage of open-ended questions,” scored higher than 3 (mean (SD) = 3.71 (1.09)). In Habit 2, only the item “eliciting the patient's understanding of the problem” (mean (SD) = 3.24 (1.38)) scored higher than 3. Four of the items in Habit 4 scored higher than the 3: “explaining using the patient's frame of reference” (mean (SD) = 3.71 (1.23)), “giving clear explanations” (mean (SD) = 3.34 (1.30)), “explaining the rationale for tests” (mean (SD) = 3.76 (1.26)), and “encouraging additional questions” (mean (SD) = 3.32 (1.77)).

**Table 3:** Scores of four habits coding scheme.

Four Habits Coding Scheme^a^	Baseline Mean score (SD) N = 38	Pre-test score, N=23^b^		Post-test score, N=23^b^		*Z*-statistics c	*p*-value ^c^
		Median (IQR)	Mean (SD)	Median (IQR)	Mean (SD)		
Habit 1: Invest in the Beginning							
1A: Greet warmly	1.87 (1.04)	2 (1–3)	1.96 (1.02)	3 (1–5)	3.17 (1.61)	−2.77	0.006⁎
1B: Engage in small talk	1.29 (0.70)	1 (1–1)	1.26 (0.69)	2 (1–3)	2.00 (1.24)	−2.71	0.007⁎
1C: Use mainly open-ended questions	3.71 (1.09)	4 (3–5)	3.87 (1.01)	3 (3–5)	3.52 (1.34)	−1.15	0.25
1D: Expansion of concerns	2.71 (1.47)	3 (1–4)	2.74 (1.48)	4 (3–5)	3.78 (1.28)	−2.73	0.006⁎
1E: Elicit full agenda	2.76 (1.15)	3 (2–3)	2.83 (1.15)	3 (3–4)	3.22 (1.17)	−1.62	0.106

Habit 2: Elicit Patient's Perspective							
2A: Patient's understanding of the problem	3.24 (1.38)	3 (2–4)	3.04 (1.36)	3 (3–5)	3.61 (1.20)	−1.56	0.118
2B: Ask patient's goals for visit	2.16 (1.26)	2 (1–3)	2.09 (1.16)	3 (2–4)	2.96 (1.22)	−2.37	0.018⁎
2C: Assess impact on life	2.79 (1.40)	2 (2–4)	2.65 (1.34)	4 (2–4)	3.70 (1.11)	−2.91	0.004⁎

Habit 3: Demonstrate Empathy							
3A: Encourage emotional expression	1.74 (0.98)	1 (1–3)	1.91 (1.08)	2 (1–3)	1.96 (0.93)	−0.37	0.715
3B: Accept/validate patient's feelings	2.00 (1.34)	1 (1–4)	2.13 (1.46)	3 (2–3)	2.52 (0.85)	−1.31	0.192
3C: Help to identify feelings	1.76 (0.97)	1 (1–3)	1.91 (1.12)	3 (2–3)	2.61 (0.94)	−2.27	0.023⁎

Habit 4: Invest in the End							
4A: Use patient's frame of reference	3.71 (1.23)	3 (3–5)	3.61 (1.20)	3 (3–5)	3.48 (1.08)	−0.60	0.549
4B: Allow time for information to be absorbed	2.84 (1.17)	3 (2–3)	2.74 (1.05)	3 (3–3)	3.13 (0.97)	−1.86	0.063
4C: Give clear explanations	3.34 (1.30)	3 (3–5)	3.43 (1.27)	3 (3–5)	3.65 (1.07)	−0.43	0.667
4D: Explain rationale for tests	3.76 (1.26)	3 (3–5)	3.57 (1.27)	4 (3–5)	3.78 (1.28)	−0.65	0.517
4E: Test patient's comprehension	2.79 (1.23)	3 (1–3)	2.43 (1.27)	3 (2–3)	2.65 (1.07)	−1.02	0.308
4F: Encourage involvement in decision-making	1.71 (1.18)	2 (1–2)	1.91 (1.20)	2 (1–3)	2.04 (1.26)	−0.35	0.723
4G: Explore acceptability of treatment plan	2.87 (1.44)	3 (1–4)	2.61 (1.41)	4 (3–5)	3.74 (1.45)	−2.58	0.01⁎
4H: Explore barriers to implement treatment	2.63 (1.36)	3 (1–3)	2.48 (1.44)	4 (3–5)	3.61 (1.37)	−2.86	0.004⁎
4I: Encourage questions	3.32 (1.77)	3 (1–5)	2.91 (1.78)	5 (1–5)	3.56 (1.73)	−1.37	0.17
4J: Plan for follow-up	2.95 (1.75)	3 (1–5)	2.74 (1.71)	5 (4–5)	4.30 (1.26)	−2.70	0.007⁎

a Rated from 1 = Not very effective to 5 = Very Effective.

b Only 23 out of the 38 participants managed to complete the post-test data collection; hence, only 23 pairs of data were included for comparison.

c Wilcoxon signed-rank test performed for pre-test and post-test scores.

⁎ p < 0.05.

As explained above, only 23 participants managed to complete the post-intervention data collection; thus, only 23 complete pre- and post-intervention datasets were included in this comparison. Nonetheless, the Mann–Whitney *U* test did not reveal a significant difference in the baseline score between the 15 withdrawn participants and 23 participants who completed the post-intervention data collection (*p* = 0.26).

The Wilcoxon signed-rank test was performed for the pre- and post-intervention scores (*n* = 23). Overall, nine items in the FHCS showed a significant increase from before the communication training (Table 3).

In Habit 1, “invest in the beginning,” three items showed a significant increase in scores. The items were 1A, “greet warmly” (pre-test median = 2, post-test median = 3, Z = −2.77); 1B, “engaging in small talk” (pre-test median = 1, post-test median = 2, Z = −2.71); and 1D, “expansion of concerns” (pre-test median = 3, post-test median = 4, Z = −2.73; *p* < 0.05). This indicates that there was a significant increase in instances of small talk and expansion of patients' concerns, such as exploring patients' issues of not injecting insulin.

In Habit 2, “elicit patients' perspectives,” two of the three items showed significant changes: 2B, “ask about patients' goals” (pre-test median = 2, post-test median = 3, Z = −2.37) and 2C, “assess the impact on life” (pre-test median = 2, post-test median = 4, Z = −2.91; *p* < 0.05). Pharmacists showed increased attempts to understand patients' psychosocial status in further detail, such as whether the patient was living with another family member and how their working life may affect their medication-taking routine.

In Habit 3, “demonstrate empathy,” only item 3C, “help to identify feelings” (pre-test median = 1, post-test median = 3, Z = −2.27; *p* < 0.05), showed a significant increase. This indicates that there was an increase in attempts from pharmacists' responses that they understood patients' worries and concerns about their medications, including potential side effects and difficulties in medication administration.

In Habit 4, “invest in the end,” three items were found to show a significant increase: 4G, “explore acceptability of treatment plan” (pre-test median = 3, post-test median = 4, Z = −2.58); 4H, “explore barriers to implementing treatment” (pre-test median = 3, post-test median = 4, Z = −2.86); and 4 J, “plan for follow-up” (pre-test median = 3, post-test median = 5, Z = −2.70; *p* < 0.05). When compared to the pre-intervention data, pharmacists showed an increase in attempts to ask the patients whether they were satisfied with the current treatment plan and also provided suggestions on how to improve adherence (e.g., setting alarms and using a pillbox).

### 3.3 Attitudes toward concordance (RUS-LATCon)

The internal consistency of the RUS-LATCon for pre-training (α = 0.72) and post-training (α = 0.87) were found to be acceptable. Table 4 presents the mean scores of each item in the RUS-LATCon and comparisons of the scores before and after training. Overall, there was a significant difference (p < 0.05) in the pre-intervention (mean (SD) = 4.06 (0.25)) and post-intervention (mean (SD) = 4.23 (0.40)) total mean scores. Three items (items 3, 4, and 5) were found to exhibit a significant increase in scores from pre-intervention to post-intervention. Item 3 indicated that there was an increase in recognizing patients' needs, desires, and capabilities regarding the use of medications. Items 4 and 5 were related to medication uncertainty during medication-taking, such as whether patients were able to follow medication-taking directions. Increases in the scores of items 4 and 5 indicated that pharmacists demonstrated increased awareness that uncertainty about taking medications may arise during the encounter, such as the perceived effectiveness of the medications.

**Table 4:** Mean and SD of RUS-LATCon before and after training (*n* = 23).

Statement^a^	Pre-intervention score, Mean (SD)	Post-intervention score, Mean (SD)	*t*-statistic (df = 22)	*p*-value
1. During a counseling session, the pharmacist and patient should treat each other like equal partners	3.87 (0.55)	4.17 (0.65)	−1.58	0.13
2. Pharmacists should respect their patients' beliefs and coping abilities about use of medications	4.22 (0.60)	4.30 (0.70)	−0.62	0.54
3. Pharmacists should pay attention to patients' desires, needs, and capabilities about use of medications	3.87 (0.76)	4.22 (0.67)	−2.34	0.03⁎
4. The patient does not always know how they will follow the directions provided when taking medication	3.70 (0.70)	4.04 (0.48)	−2.34	0.03⁎
5. Pharmacists are hopeful but not always positive that medication prescribed will improve patient health	2.96 (0.93)	3.43 (0.99)	−2.90	0.01⁎
6. Pharmacists should ask the patient to share their ideas about how their illness should be treated	3.91 (0.73)	4.26 (0.62)	−1.79	0.09
7. Pharmacists should discuss and agree upon a treatment plan with their patients	4.35 (0.57)	4.35 (0.57)	0.00	1.000
8. Both the patient and pharmacist should agree on a plan to reach the desired effects of treatment options	4.43 (0.51)	4.48 (0.51)	−0.37	0.71
9. Pharmacists should help patients make informed decisions by giving them information about the risks and benefits of different treatments	4.39 (0.50)	4.43 (0.59)	−0.37	0.71
10. The patient's desired outcomes and willingness to follow directions is the most critical element in planning the treatment	4.48 (0.66)	4.39 (0.66)	0.57	0.58
11. During the pharmacist-patient consultation the patient's decision is the most important	4.35 (0.49)	4.35(0.57)	0.00	1.000
12. The decision to use medications should be based on what the patient wants and can achieve	3.83 (0.83)	4.04 (0.77)	−1.05	0.31
13. I believe that pharmacists should be more sensitive to how patients react to the information they give	4.17 (0.49)	4.35 (0.71)	−1.70	0.10
14. I believe pharmacists need to learn about patient's beliefs about medications	4.26 (0.54)	4.35 (0.71)	−0.53	0.60
Total mean	4.06 (0.25)	4.23 (0.40)	−2.30	0.03⁎

a Likert scale, 1 = Strongly disagree, 2 = disagree, 3 = Neutral, 4 = Agree, 5 = Strongly agree.

⁎ Significant at p < 0.05 level.

### 3.4 Self-reported retrospective pre–post communication self-efficacy

**Fig. 2:**
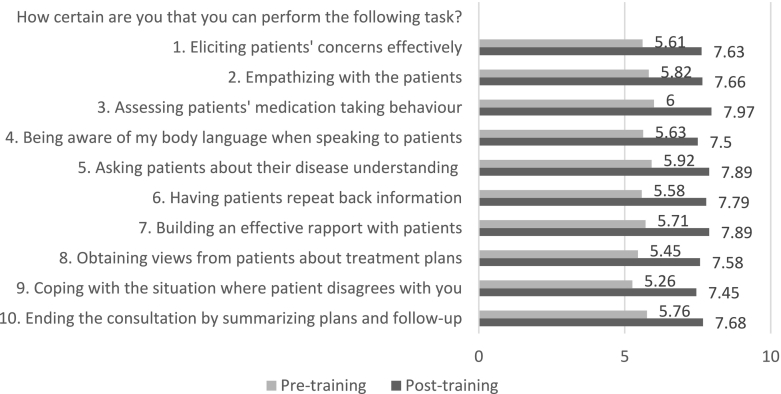
Communication Self-Efficacy Scores.

Cronbach's alpha values for pre-intervention (α = 0.97) and post-intervention (α = 0.92) communication self-efficacy data were found to be good. Fig. 2 illustrates the mean scores of communication self-efficacy for each of the ten items before and after the training. There was a significant increase in the overall mean scores from the initial retrospective scores mean (SD) = 5.67 (1.44) to the post-training scores mean (SD) = 7.71 (0.94); *p* < 0.001). There were significant changes in the mean scores for each item (*p* < 0.05), indicating that pharmacists generally felt more confident in conducting specific tasks after the training. The largest significant change was observed in the scores for item 8 (“obtaining views from patients about treatment plans”; t(37) = −11.74; p < 0.001). This was followed by the scores for item 7 (“building an effective rapport with patients”; t(37) = −11.39; p < 0.001) and item 9 (“coping with situations wherein patients disagree with you”; t(37) = −11.18; p < 0.001). These results indicated that after undergoing the training, pharmacists had more confidence in negotiating, building relationships, and involving patients in making treatment decisions during encounters.

### 3.5 Program evaluation feedback

In general, the participants were satisfied with the fulfillment of the program objectives from the sessions. In the retrospective pre–post question regarding the importance of CST for pharmacists, there was a significant increase in the score from before the training (mean (SD) = 3.84 (0.97)) to that post-training (mean (SD) = 4.73 (0.45)), t(37) = −6.63, *p* < 0.05). Based on the feedback survey, the participants acknowledged the importance of this training program for practice in MTACs and offered several constructive feedback points. The participants suggested that the training be further expanded to other types of MTACs, which may further indicate that the participants found the program to be useful.

In terms of the most useful session, most of the pharmacists found the MI session to be the most useful for practice, particularly the group practice scenario exercises. The participants reported that the MI techniques learned during this session were relevant and useful for routine patient encounters in MTACs. In addition, the participants felt that having speakers with different backgrounds (e.g., academia and hospital pharmacists) contributed to the sharing of various perspectives and experiences, which improved their learning experience. The participants suggested that having a checklist or roadmap summarizing practical patient-centered communication skills may be a useful reference for practicing pharmacists in the future. There were also several suggestions on ways to improve the training program. For instance, the participants preferred more interactive practice scenarios or role-play sharing sessions. In addition, several participants also suggested video demonstrations of patient-centered approaches to facilitate understanding and learning.

## 4 Discussion

Existing studies have highlighted communication as one of the key components to implementing PCC effectively; thus, patient-centered communication training was developed and conducted for pharmacists.^51^ This study evaluated the effects of a CST intervention, based on MI and the FHM on pharmacists' attitudes toward concordance, communication self-efficacy, and patient-centered communication scores (based on the FHCS).

At baseline, pharmacists generally did well on FHCS items related to information-gathering and counseling activities, such as asking open-ended questions and providing explanations on disease and drug-related issues. This finding highlights the fact that pharmacists are well versed in providing education to patients, which is one of the major MTAC activities to ensure patients' adherence to medication. However, several items were identified to have lower scores and may need to be addressed, such as asking about patients' goals and perspectives, demonstrating empathy, and encouraging involvement in decision-making.

One of the key aspects of PCC is that healthcare providers should seek to understand patients' individual goals, concerns, and perspectives regarding disease management and medication-taking.^52^ The scores of the FHCS indicated that pharmacists demonstrated a significant increase in their attempts to explore patients' perspectives and emotions after the CST, particularly in asking about patients' personal goals and addressing their concerns about the disease. Furthermore, pharmacists also demonstrated an increased score in attitudes (based on the RUS-LATCon) related to recognizing patients' desires and needs regarding medication use, along with an increased awareness of patients potentially having unaddressed medication uncertainties. This finding is consistent with a study by Luetsch et al. (2017), where pharmacists realized that their past communication was not as patient-centered as they had thought. It was only after applying patient-centered techniques like MI that they felt able to improve their interactions and build genuine interests in understanding patients' needs.^34^ This is important because studies on communication training, which focused on aspects such as eliciting patients' concerns and probing their disease understanding, have shown to improve patients' clinical outcomes.^16^^,^^53^^,^^54^ Knowing patients' concerns and disease understanding may facilitate the provision of tailored information to them, increasing mutual trust and patient health literacy.^55^ Furthermore, patients may feel heard and understood when providers take the time to listen to their disease concerns, which may also help alleviate any anxiety that patients may develop, especially when newly diagnosed with a disease.^19^

Shared decision-making (SDM) is an important part of PCC wherein decisions regarding any treatment plan are agreed upon together by the providers and patients, while also accounting for the patients' treatment preferences. This helps ensure that patients can follow through with the regimen without any unaddressed barriers or uncertainties.^56^ However, despite the positive impact of the CST on pharmacists' attitudes and communication skills in terms of addressing any underlying issues, there were no significant post-training changes in the score for involving patients in decision-making, such as encouraging patients to voice their preferences or inputs in the treatment decision-making process. Nonetheless, the post-training FHCS scores revealed increased efforts to understand patients' disease concerns and explore barriers and acceptability of treatment for patients. Even though the CST showed positive effects in enhancing patient-centered communication, further improvements are required to effectively involve patients in the decision-making process. One possible explanation for the lack of improvement may be the inadequate focus on the topic of SDM during the CST. A recent study found that an interprofessional shared decision-making program effectively improved the competency, self-efficacy and intention of multidisciplinary healthcare personnel to engage in SDM.^57^ Thus, it is recommended that future communication studies focus more on practical approaches toward SDM. For example, the intervention may include a validated SDM competency framework to guide teaching, as well as practical guidance on the use of patient decision aids to facilitate SDM.^58^^,^^59^

Demonstrating empathy is one of the key components of the FHM, and it has been suggested to contribute significantly to building patient–provider relationships and improving patient satisfaction. Patients tend to feel more supported and are more inclined to share their personal concerns and information with their providers when empathy is effectively expressed.^60^, ^61^, ^62^, ^63^ However, findings from this study showed that despite the increased attempts to identify patients' feelings, there were no significant changes in other items related to empathy. This contrasted with another study among student pharmacists that found improvements in several items related to empathy including encouraging emotional expression and good non-verbal behaviors.^26^ However, the authors did note that time constraints could limit the extent of empathy that can be portrayed during an encounter.^26^ One possible explanation for the findings in our study is that the CST program, although incorporating elements of empathy through the MI and the FHM frameworks, might not have adequately conveyed strategies to address emotional needs. Additionally, evaluating empathy solely based on audio recordings alone may be challenging, as empathy is often expressed non-verbally through facial expressions or body gestures.^64^ Moreover, the emotional aspects in the SP case scenario may not have been sufficiently highlighted, potentially influencing the expression of emotional needs during the simulated consultation. Nevertheless, future research should focus on addressing the emotional needs of patients, such as demonstrating specific communication practice techniques or through video demonstration. A systematic review also found that learning empathy through simulation-based education may be effective. Simulation-based education may consist of teaching through role-play with SPs and providing feedback after, or even requesting SPs to share their knowledge and experience on empathy.^65^

Findings from this study revealed an increase in participants' self-efficacy in completing patient-centered communication tasks after the training, consistent with findings from other studies.^14^^,^^15^^,^^47^^,^^61^^,^^66^ Although it is not a direct measure of patient-centered communication competency, this increase in self-efficacy may indicate the adaptability and effectiveness of the training in encouraging pharmacists to implement these skills in their practice.^66^^,^^67^ As such, higher self-efficacy may indicate increased confidence and motivation of an individual to engage in the learned communication behaviors in real-life encounters.^17^^,^^68^ This is supported by a previous study, whereby self-efficacy was significantly associated with improved patient-centered communication performance.^69^ Therefore, this underlines the importance of communication self-efficacy in ensuring the translation of the skills to practice.

Based on previous studies, women were expected to score significantly higher than men in patient-centered communication.^28^^,^^70^, ^71^, ^72^ Studies have often highlighted that women tend to demonstrate more patient-centered communication tasks such as showing empathy and exploring patients' concerns.^28^^,^^70^, ^71^, ^72^, ^73^ Interestingly, in this study, men scored significantly higher than women at baseline. However, it may be possible that the substantial difference in the number of male and female participants skewed the data.

The CST program generally received positive feedback from the participants, with the MI session being the most well-received part of the training. The participants also commented that the sharing sessions by speakers with various backgrounds (e.g., academicians, hospital practitioners, and dieticians) were useful and improved the learning experience. Nonetheless, in the feedback questionnaire, the participants suggested including more practice scenarios or role-play sessions and videos demonstrating patient-centered skills. This may explain why participants rated the MI session highly, as this was the only session that included a hands-on approach with practice scenarios. Studies have suggested that participants who undergo experiential learning, including role-play or practice scenarios, demonstrate a better understanding of the taught skills and concepts.^34^^,^^74^^,^^75^ Difficult-to-grasp or unfamiliar subjects are generally better taught through experiential learning, such as role-play or case-scenario discussions.^76^^,^^77^ Therefore, future CST programs are recommended to include more interactive sessions to maximize the learning experience, facilitating the translation of skills to practice.

### 4.1 Suitability of the FHM to the pharmacy context

There are a limited number of studies that have adapted the FHM into pharmacy practice; some studies have adapted it into the pharmacy student curriculum.^26^ Overall, the FHCS is a straightforward, easy-to-use tool to measure patient-centered communication in patient encounters. However, since this coding scheme was originally developed for physician consultations, there are undoubtedly some differences that must be addressed when it is adapted to the pharmacist MTAC setting.^20^^,^^78^ For example, from the codebook, the item “elicit full agenda” describes repeatedly asking patients to explain the full range of their symptoms before arriving at a diagnosis, something that may not be relevant in MTACs as the preliminary aim of MTAC is to detect and solve medication adherence related problems. However, in the present study, this criterion was scored based on pharmacists prompting further on any additional concerns to be addressed based on patients' current medications instead. Agreeing with the remark by Grice et al. (2013), some items were also subjective. For example, the highest score criterion for “small talk” is described as “making non-medical comments to put patients at ease.” As such, it can be subjective to score this criterion, as cursory attempts at small talk may not necessarily create rapport or put the patients at ease.^26^ Nonetheless, the FHM (and its corresponding scoring rubric, the FHCS) was useful in the evaluation of patient-centered communication in pharmacist MTAC encounters. Revisions may be needed to further adapt this tool to the pharmacy context, particularly in MTACs, including adapting the descriptions for the scoring criteria to be more relevant to pharmacy practice, such as for the item “elicit full agenda,” as described above.

### 4.2 Limitations

To the authors' knowledge, this was the first study in Malaysia to adopt the validated FHM and MI in CST among pharmacists from different tertiary hospitals and to utilize SPs to evaluate its impact. However, several limitations of this study must be acknowledged. First, there was a lack of randomization and a control group. This limited data interpretation and inferences that could be drawn from the differences in the pre- and post-intervention scores. Furthermore, the study design may have introduced bias, including subject bias and maturation (whereby given time, the participants would have exhibited an improvement anyway). However, post-test data collection was performed within <2 weeks after the training, which may have reduced the maturation effect.

Second, as this study utilized SPs to measure patient-centered communication in encounters, the results may not be reflected in real-life clinical practice with real patients. For example, the behavior of SPs may be more interactive or approachable than that of regular patients; thus, they might not be representative of different patients' behaviors (e.g., passiveness or aggressiveness). Additionally, certain aspects of communication, such as empathy conveyed by non-verbal gestures, may have been missed as pharmacists' non-verbal behaviors were not assessed. Although the CST in the present study revealed evidence of improvements in communication self-efficacy as well as patient-centered communication scores from the simulated consultations, for future recommendations, it may be worthwhile to measure the impact of CST in real-life encounters. Nevertheless, the use of SPs may be advantageous in portraying consistent scenarios with high degrees of reproducibility, which minimizes variations in the data.^42^ Another advantage is that using experienced SPs does not cause any direct risk to real patients, especially when evaluating newly acquired skills such as communication skills.^42^

The present study also recruited only two MTAC types, namely diabetes and warfarin management, which may limit its generalizability to different MTACs. Thus, it may be useful to determine how patient-centered communication training may be applied to other types of MTACs, such as human immunodeficiency virus and psychiatry MTACs. As such, future studies may consider testing patient-centered CST in other fields to evaluate its suitability and benefits to the practice, such as in the community pharmacy setting.

Another limitation was the substantial number of dropouts due to the Movement Control Order caused by the COVID pandemic in post-training data collection. After extensive consideration, the 15 participants were excluded as the difference in the timeline in collecting the post-intervention data might have been substantially different from that of the 23 participants who had completed the intervention earlier, which may have led to inconsistent findings. However, this may have caused some bias in the post-training data, which may have altered the true outcomes to a certain extent. Nonetheless, 23 participants met the minimum sample size with adequate statistical power.

## 5 Conclusions

Findings from this study suggest that CST based on patient-centered communication frameworks, such as the FHM and MI, has the potential to improve patient-centered communication scores, attitudes toward concordance, and communication self-efficacy of pharmacists. Positive self-efficacy may increase the chances of pharmacists attempting to practice patient-centered skills in encounters. Recommendations are made to further enhance and tailor patient-centered communication models into the pharmacy context to maximize the benefits that the models can offer to practice.

## Funding

This study was funded by a research grant from Universiti Kebangsaan Malaysia (code: GUP-2018-139).

## Declaration of Competing Interest

The authors declare that they have no known competing financial interests or personal relationships that could have appeared to influence the work reported in this paper.

## Appendix A Supplementary data

List of Abbreviations Image 1

## References

[1] Grice G.R., Gattas N.M., Prosser T. (2017). Design and validation of patient-centered communication tools (PaCT) to measure students’ communication skills. Am J Pharm Educ.

[2] Rust C., Gentry W.M., Ford H. (2019). Assessment of the effect of communication skills training on communication apprehension in first year pharmacy students – a two-year study. Curr Pharm Teach Learn..

[3] Thamby S.A., Subramani P. (2014). Seven-star pharmacist concept by World Health Organization. J Young Pharm.

[4] Stevenson F.A., Cox K., Britten N., Dundar Y. (2004). A systematic review of the research on communication between patients and health care professionals about medicines: the consequences for concordance. Health Expect.

[5] Capone V. (2014). Patient communication self-efficacy, self-reported illness symptoms, physician communication style and mental health and illness in hospital outpatients. J Health Psychol.

[6] Bachmann C., Barzel A., Roschlaub S., Ehrhardt M., Scherer M. (2013). Can a brief two-hour interdisciplinary communication skills training be successful in undergraduate medical education?. Patient Educ Couns.

[7] Sherwood A., Brinkmann J., Fatone S. (2018). Review of benefits to practitioners of using good patient-practitioner communication. J Prosthetics Orthot.

[8] Scholl I., Zill J.M., Härter M., Dirmaier J. (2014). An integrative model of patient-centeredness - a systematic review and concept analysis. PLoS One.

[9] Brown M., Bussell J. (2011). Medication adherence: WHO cares?. Mayo Clin Proc.

[10] King A., Hoppe R.B. (2013). “Best practice” for patient-centered communication: a narrative review. J Grad Med Educ.

[11] Chong W.W., Aslani P., Chen T.F. (2014). Pharmacist-patient communication on use of antidepressants: a simulated patient study in community pharmacy. Res Social Adm Pharm.

[12] Kwame A., Petrucka P.M. (2021). A literature-based study of patient-centered care and communication in nurse-patient interactions: barriers, facilitators, and the way forward. BMC Nurs.

[13] Helitzer D.L., LaNoue M., Wilson B., de Hernandez B.U., Warner T., Roter D. (2011). A randomized controlled trial of communication training with primary care providers to improve patient-centeredness and health risk communication. Patient Educ Couns.

[14] Birgitte N., Jette A., Ohm Kyvik K., Kofoed P.-E. (2012). Communication skills training increases self-efficacy of health care professionals. J Contin Educ Heal Prof.

[15] Saslaw M., Sirota D.R., Jones D.P. (2017). Effects of a hospital-wide physician communication skills training workshop on self-efficacy, attitudes and behavior. Patient Exp J.

[16] Cooper L.A., Roter D.L., Carson K.A. (2011). A randomized trial to improve patient-centered care and hypertension control in underserved primary care patients. J Gen Intern Med.

[17] Hagemeier N.E., Hess R., Hagen K.S., Sorah E.L. (2014). Impact of an interprofessional communication course on nursing, medical, and pharmacy students’ communication skill self-efficacy beliefs. Am J Pharm Educ.

[18] Liekens S., Vandael E., Roter D. (2014). Impact of training on pharmacists’ counseling of patients starting antidepressant therapy. Patient Educ Couns.

[19] Hanya M., Kanno Y., Akasaki J., Abe K., Fujisaki K., Kamei H. (2017). Effects of communication skill training (CST) based on SPIKES for insurance-covered pharmacy pharmacists to interact with simulated cancer patients. J Pharm Heal Care Sci.

[20] Frankel R.M., Stein T. (1999). Getting the most out of the clinical encounter: the four habits model. J Med Pract Manage.

[21] Greenhill N., Anderson C., Avery A., Pilnick A. (2011). Analysis of pharmacist-patient communication using the Calgary-Cambridge guide. Patient Educ Couns.

[22] Braverman A.M., Kunkel E.J., Katz L. (2015). Do I buy it? How AIDETTM training changes residents’ values about patient care. Journal of Patient Experience.

[23] Lonsdale C., Hall A.M., Williams G.C. (2012). Communication style and exercise compliance in physiotherapy (CONNECT). A cluster randomized controlled trial to test a theory-based intervention to increase chronic low back pain patients’ adherence to physiotherapists’ recommendations: study rationale, design, and methods. BMC Musculoskelet Disord.

[24] Brust-Sisti L.A., Sturgill M., Volino L.R. (2019). Situation, background, assessment, recommendation (SBAR) technique education enhances pharmacy student communication ability and confidence. Curr Pharm Teach Learn.

[25] Naughton C. (2018). Patient-centered communication. Br Med J.

[26] Grice G.R., Gattas N.M., Sailors J. (2013). Health literacy: use of the four habits model to improve student pharmacists’ communication. Patient Educ Couns.

[27] Ford C.R., Garza K., Kavookjian J., Kleppinger E.L. (2019). Assessing student pharmacist communication skills: development and implementation of a communication rubric. Curr Pharm Teach Learn..

[28] Fossli Jensen B., Gulbrandsen P., Dahl F.A., Krupat E., Frankel R.M., Finset A. (2011). Effectiveness of a short course in clinical communication skills for hospital doctors: results of a crossover randomized controlled trial (ISRCTN22153332). Patient Educ Couns.

[29] Krupat E., Frankel R., Stein T., Irish J. (2006). The four habits coding scheme: validation of an instrument to assess clinicians’ communication behavior. Patient Educ Couns.

[30] Rosengren D.B. (2017). A Practitioner Workbook.

[31] Miller W.R., Rollnick S. (2012). https://books.google.com.my/books?id=IuTbPPZUTjwC.

[32] Steinberg M.P., Miller W.R. (2015). https://books.google.com.my/books?id=D-EyCgAAQBAJ.

[33] Jaffray M., Matheson C., Bond C.M. (2014). Does training in motivational interviewing for community pharmacists improve outcomes for methadone patients? A cluster randomised controlled trial. Int J Pharm Pract.

[34] Luetsch K., Burrows J. (2018). From transitions to transformation – a study of pharmacists developing patient-centered communication skills. Res Soc Adm Pharm.

[35] Duff A., Latchford G. (2016). Using motivational interviewing to improve medicines adherence. Pharm J.

[36] Ministry of Health (2013). Perkhidmatan medication therapy adherence clinic (MTAC). Pharmaceutical Services Division.

[37] Ng Y.K., Shah N.M., Loong L.S., Pee L.T., Hidzir S.A.M., Chong W.W. (2018). Attitudes toward concordance and self-efficacy in decision making: a cross-sectional study on pharmacist-patient consultations. Patient Prefer Adherence.

[38] Rajah R., Hassali M.A., Lim C.J. (2018). An interprofessional evaluation of health literacy communication practices of physicians, pharmacists, and nurses at public hospitals in Penang, Malaysia. Ann Pharmacother.

[39] Awaisu A., Abd Rahman N.S., Nik Mohamed M.H., Bux S.H.B.R., Nazar N.I.M. (2010). Malaysian pharmacy students’ assessment of an objective structured clinical examination (OSCE). Am J Pharm Educ.

[40] Faul F., Erdfelder E., Lang A.-G., Buchner A. (2007). G * Power 3 : A flexible statistical power analysis program for the social , behavioral , and biomedical sciences. Behav Res Methods.

[41] Brown R.F., Bylund C.L., Gueguen J.A., Diamond C., Eddington J., Kissane D. (2010). Developing patient-centered communication skills training for oncologists: describing the content and efficacy of training. Commun Educ.

[42] Collins J.C., Chong W.W., de Almeida Neto A.C., Moles R.J., Schneider C.R. (2021). The simulated patient method: design and application in health services research. Res Social Adm Pharm..

[43] Bosworth H.B., Fortmann S.P., Kuntz J. (2017). Recommendations for providers on person-centered approaches to assess and improve medication adherence. J Gen Intern Med.

[44] Flagg A.J. (2010).

[45] Marinker M., Blekinsopp A., Bond C. (1997).

[46] Bandura A. (1997).

[47] Ammentorp J., Sabroe S., Kofoed P.E., Mainz J. (2007). The effect of training in communication skills on medical doctors’ and nurses’ self-efficacy: a randomized controlled trial. Patient Educ Couns.

[48] Parle M., Maguire P., Heaven C. (1997). The development of a training model to improve health professionals’ skills, self-efficacy and outcome expectancies when communicating with cancer patients. Soc Sci Med.

[49] Bhanji F., Gottesman R., De Grave W., Steinert Y., Winer L.R. (2012). The retrospective pre-post: a practical method to evaluate learning from an educational program. Acad Emerg Med.

[50] Drennan J., Hyde A. (2008). Controlling response shift bias: the use of the retrospective pre-test design in the evaluation of a master’s programme. Assess Eval High Educ.

[51] Ng Y.K., Shah N.M., Loong L.S., Pee L.T., Chong W.W. (2019). Patient-centred care in the context of pharmacy consultations : a qualitative study with patients and pharmacists in Malaysia. J Eval Clin Pract.

[52] Hong H., Oh H.J. (2020). The effects of patient-centered communication: exploring the mediating role of trust in healthcare providers. Health Commun.

[53] Lonsdale C., Hall A.M., Murray A. (2017). Communication skills training for practitioners to increase patient adherence to home-based rehabilitation for chronic low back pain: results of a cluster randomized controlled trial. Arch Phys Med Rehabil.

[54] Ryan B.L., Brown J.B., Tremblay P.F., Stewart M. (2019). Measuring patients’ perceptions of health care encounters: examining the factor structure of the revised patient perception of patient-centeredness (PPPC-R) questionnaire. J Patient-Centered Res Rev.

[55] Ruben M.A., Blanch-Hartigan D., Hall J.A. (2020). The Wiley Handbook of Healthcare Treatment Engagement.

[56] Stalnikowicz R., Brezis M. (2020). Meaningful shared decision-making: complex process demanding cognitive and emotional skills. J Eval Clin Pract.

[57] Hsiao C.Y., Wu J.C., Lin P.C. (2022). Effectiveness of interprofessional shared decision-making training: a mixed-method study. Patient Educ Couns.

[58] Tong W.T., Ng C.J., Lee Y.K., Lee P.Y. (2020). What will make patients use a patient decision aid? A qualitative study on patients’ perspectives on implementation barriers and facilitators. J Eval Clin Pract.

[59] Aljumah K., Hassali M.A. (2015). Impact of pharmacist intervention on adherence and measurable patient outcomes among depressed patients : a randomised controlled study. BMC Psychiatry.

[60] Andersen F.A., Johansen A.S.B., Søndergaard J., Andersen C.M., Assing Hvidt E. (2020). Revisiting the trajectory of medical students’ empathy, and impact of gender, specialty preferences and nationality: a systematic review. BMC Med Educ.

[61] Kerr J.L., Stahnke A.M., Behnen E.M. (2015). Assessing empathy and self-efficacy levels of pharmacy students in an elective diabetes management course. Am J Pharm Educ.

[62] Grayson-Sneed K.A., Dwamena F.C., Smith S., Laird-Fick H.S., Freilich L., Smith R.C. (2016). A questionnaire identifying four key components of patient satisfaction with physician communication. Patient Educ Couns.

[63] Maxfield H., Delzell J.E., Chumley H. (2011). Eliciting the patient’s perspective: does experience or type of case make a difference?. Patient Educ Couns.

[64] Laughey W., Sangvik Grandal N., Stockbridge C., Finn G.M. (2019). Twelve tips for teaching empathy using simulated patients. Med Teach.

[65] Bearman M., Palermo C., Allen L.M., Williams B. (2015). Learning empathy through simulation: a systematic literature review. Simul Healthc.

[66] Grome L.J., Banuelos R.C., Lopez M.A., Nicome R.K., Leaming-Van Zandt K.J. (2018). Communication course for pediatric providers improves self-efficacy. Plast Reconstr Surg - Glob Open.

[67] Boling A.M. (2020).

[68] Rogers E.R., King S.R. (2012). The influence of a patient-counseling course on the communication apprehension, outcome expectations, and self-efficacy of first-year pharmacy students. Am J Pharm Educ.

[69] Gulbrandsen P., Jensen B.F., Finset A., Blanch-Hartigan D. (2013). Long-term effect of communication training on the relationship between physicians’ self-efficacy and performance. Patient Educ Couns.

[70] Noble L.M., Kubacki A., Martin J., Lloyd M. (2007). The effect of professional skills training on patient-centredness and confidence in communicating with patients. Med Educ.

[71] Roter D.L., Hall J.A. (2004). Physician gender and patient-centered communication: a critical review of empirical research. Annu Rev Public Health.

[72] Jackson J.L., Farkas A., Scholcoff C. (2020). Does provider gender affect the quality of primary care?. J Gen Intern Med.

[73] Shin D.W., Roter D.L., Roh Y.K., Hahm S.K., Cho B.L., Park H.K. (2015). Physician gender and patient centered communication: the moderating effect of psychosocial and biomedical case characteristics. Patient Educ Couns.

[74] Uhm J., Ko Y., Kim S. (2019). Nurse education today implementation of an SBAR communication program based on experiential learning theory in a pediatric nursing practicum : a quasi-experimental study. Nurse Educ Today.

[75] Brown D.W., Atwood T.F., Moore K.L. (2018). A program to train medical physicists for direct patient care responsibilities. J Appl Clin Med Phys.

[76] Karnieli-Miller O., Michael K., Segal O., Steinberger A. (2018). Assessing an intervention focused on enhancing interpersonal communication skills and humor: a multi-method quasi-experiential study among medical students. Health Commun.

[77] Hsu L.L., Huang Y.H., Hsieh S.I. (2014). The effects of scenario-based communication training on nurses’ communication competence and self-efficacy and myocardial infarction knowledge. Patient Educ Couns.

[78] Stein T., Frankel R.M., Krupat E. (2005). Enhancing clinician communication skills in a large healthcare organization: a longitudinal case study. Patient Educ Couns.

