# Supplementary material for: Impact of a training program on hospital pharmacists' patient-centered communication attitudes and behaviors
Source: Explor Res Clin Soc Pharm. 2023 Aug 26;11:100325. doi: 10.1016/j.rcsop.2023.100325 (PMC10485631; doi:10.1016/j.rcsop.2023.100325)
Supplement: Supplementary file 1 — List of Abbreviations [file mmc1.docx]

**Appendix 1**

List of abbreviations

| COVID-19  CST  FHCS  FHM  LATCon  MCO  MI  MTAC  OARS  PCC  SDM  SP  SPSS  WHO | Coronavirus disease 2019  Communication skills training  Four Habits Coding Scheme  Four Habits Model  Leeds Attitudes toward Concordance  Movement Control Order  Motivational Interviewing  Medication Therapy Adherence Clinic  Open-ended questions, affirmations, reflection, summary  Patient-centered care  Shared decision-making  Simulated patients  Statistical Product and Service Solutions  World Health Organization |
| --- | --- |

 
